# Multidisciplinary treatment for infective endocarditis complicated with systemic multiple abscess due to Panton-Valentine leukocidin producing community-acquired methicillin-resistant *Staphylococcus aureus* and *Candida albicans*: a case report

**DOI:** 10.1186/s44215-023-00057-y

**Published:** 2023-07-27

**Authors:** Kojiro Terata, Shunsuke Saito, Ken Niitsuma, Misako Ohkusu, Noriko Takeuchi, Naruhiko Ishiwada, Taiki Matsuoka, Shotaro Hirota, Shohei Yokoyama, Yasuyuki Kanno, Yuta Kanazawa, Masahiro Tezuka, Yusuke Takei, Go Tsuchiya, Taisuke Konishi, Ikuko Shibasaki, Koji Ogata, Hirotsugu Fukuda

**Affiliations:** 1 Department of Cardiac and Vascular Surgery, Dokkyo Medical University, 880 Kitakobayashi, Mibu, Tochigi, 321-0293 Japan; 2 Department of Infectious Diseases, Medical Mycology Research Center, Chiba University, Chiba, Japan

**Keywords:** Infective endocarditis, Community-acquired *methicillin-resistant Staphylococcus aureus*, Panton-Valentine leukocidin

## Abstract

**Background:**

Infective endocarditis resulting from community-acquired methicillin-resistant *Staphylococcus aureus* (CA-MRSA) is a rare, but often fatal heart disorder. Here, we report a case of multidisciplinary treatment for infective endocarditis with systemic multiple abscesses due to Panton-Valentine leukocidin (PVL) producing CA-MRSA and *Candida albicans *.

**Case presentation:**

The patient suffered from infective endocarditis, destructive thyroiditis, hemorrhagic cerebral infarction due to mycotic embolism, lung abscess, multiple skeletal muscle abscess, and disseminate intravascular coagulopathy. Aggressive medical treatment as well as mechanical circulatory support was required before the curative surgical treatment. Blood cultures were positive for MRSA and *Candida albicans*. Genomic analysis of MRSA revealed Staphylococcal Cassette Chromosome mec IVc and also the virulence gene encoding PVL.

**Conclusions:**

CA-MRSA strains have higher pathogenicity and are more destructive to tissue than healthcare-associated MRSA strains because of the toxins they produce, including PVL. Multidisciplinary treatment including aggressive surgery was required to rescue the patient.

## Background

According to the epidemiological criteria established by the Centers for Disease Control and Prevention, community-acquired methicillin-resistant *Staphylococcus aureus* (CA-MRSA) infection is defined as MRSA infection in a person who has none of the following established risk factors for healthcare-associated MRSA (HA-MRSA) infection: first isolation of MRSA more than 48 h after hospital admission; history of hospitalization, surgery, dialysis, or residence in a long-term care facility within the previous year; the presence of an indwelling catheter or a percutaneous device at the time of culture; or previous isolation of MRSA [1, 2]. Panton Valentine leukocidin (PVL) is considered one of the important virulence factors of *S. aureus*. PVL is responsible for destruction of white blood cells, necrosis, and apoptosis and as a marker of CA-MRSA. Herein, we report a case of multidisciplinary treatment for infective endocarditis with systemic multiple abscesses due to the coinfection of CA-MRSA and *Candida albicans*.

## Case presentation

A 39-year-old woman with no medical history of immunosuppression, no previous hospitalization, and without any medication except an antidepressant, was admitted to a local hospital because of high fever and difficulty in body movement for 2 days. Anterior neck swelling was apparent, and laboratory data showed thyroid storm, acute renal failure, and rhabdomyolysis. She was transferred to our institution for intensive care. Her physical findings on arrival included high body temperature of 40 °C, systolic heart murmur, and Osler nodules on her fingers and toes. She was in cold and wet cardiogenic shock status with systolic blood pressure of 70 mmHg, heart rate 140/min, and respiratory rate 45/min. She was intubated, and mechanical circulatory support with venoarterial extracorporeal membrane oxygenation (ECMO) and intra-aortic balloon pumping (IABP) was immediately established for rescue. Laboratory data showed acute renal failure (creatinine 4.2 mg/dL, estimated glomerular filtration rate 10.4 ml/min/1.73 m^2^), hyperthyroidism (free thyroxine 8.3 ng/mL, free triiodothyronine 10.8 pg/mL, and thyroid stimulating hormone 0.02 μIU/mL), and signs of disseminated intravascular coagulation (platelet 2.0 × 10^4^/µL and D-dimer ≥ 30 μg/mL). Blood cultures grew MRSA and *C. albicans*. Continuous hemodiafiltration was also induced.

**Fig. 1:**
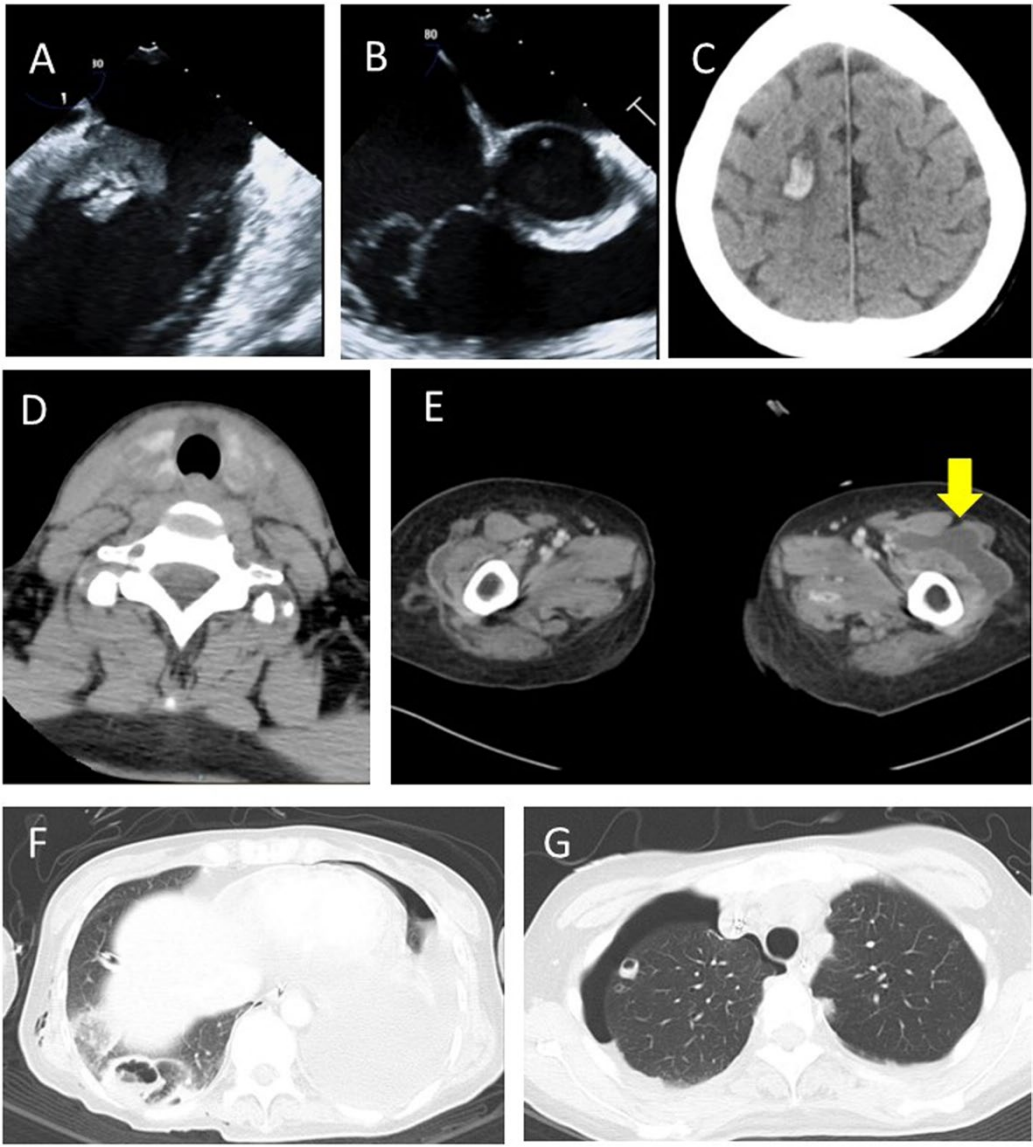
**A** 22 × 10 mm mobile mass on the posterior leaflet of the mitral valve (transesophageal echocardiography [TEE]). **B** Mobile mass on the posterior leaflet and the chordae tendineae of the tricuspid valve (TEE). **C** Hemorrhagic cerebral infarction (Brain computed tomography [CT]). **D** Bilateral thyroiditis (contrast-enhanced CT). **E** Abscess in the left thigh (contrast-enhanced CT). **F** Abscess in the right lung (chest CT). **G** Secondary pneumothorax in the right lung (chest CT)

**Fig. 2:**
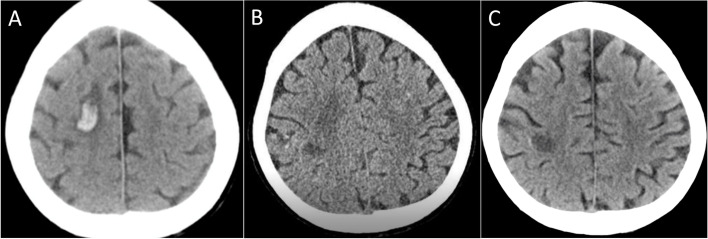
Changes in brain computed tomography (CT) findings. **A** On admission showing hemorrhagic cerebral infarction. **B** Before operation showing the regression of cerebral hemorrhage. **C** Before discharge showing no recurrence of cerebral hemorrhage

Transthoracic echocardiography showed that left ventricular wall motion was generally reduced, and its ejection fraction was 25%. Transesophageal echocardiography revealed a 22 × 10 mm mobile mass on the posterior leaflet of the mitral valve (Fig. 1A) and mild mitral regurgitation. There was also a mobile mass on the posterior leaflet and the chordae tendineae of the tricuspid valve (Fig. 1B), and tricuspid regurgitation was moderate. Brain computed tomography (CT) scan revealed hemorrhagic cerebral infarction (Fig. 1C, Fig. 2A). Contrast-enhanced CT confirmed bilateral thyroiditis (Fig. 1D); abscess formation in the left thigh (Fig. 1E), right thigh, right shoulder joints, and right lung (Fig. 1F); and secondary pneumothorax in the right lung (Fig. 1G).

**Fig. 3:**
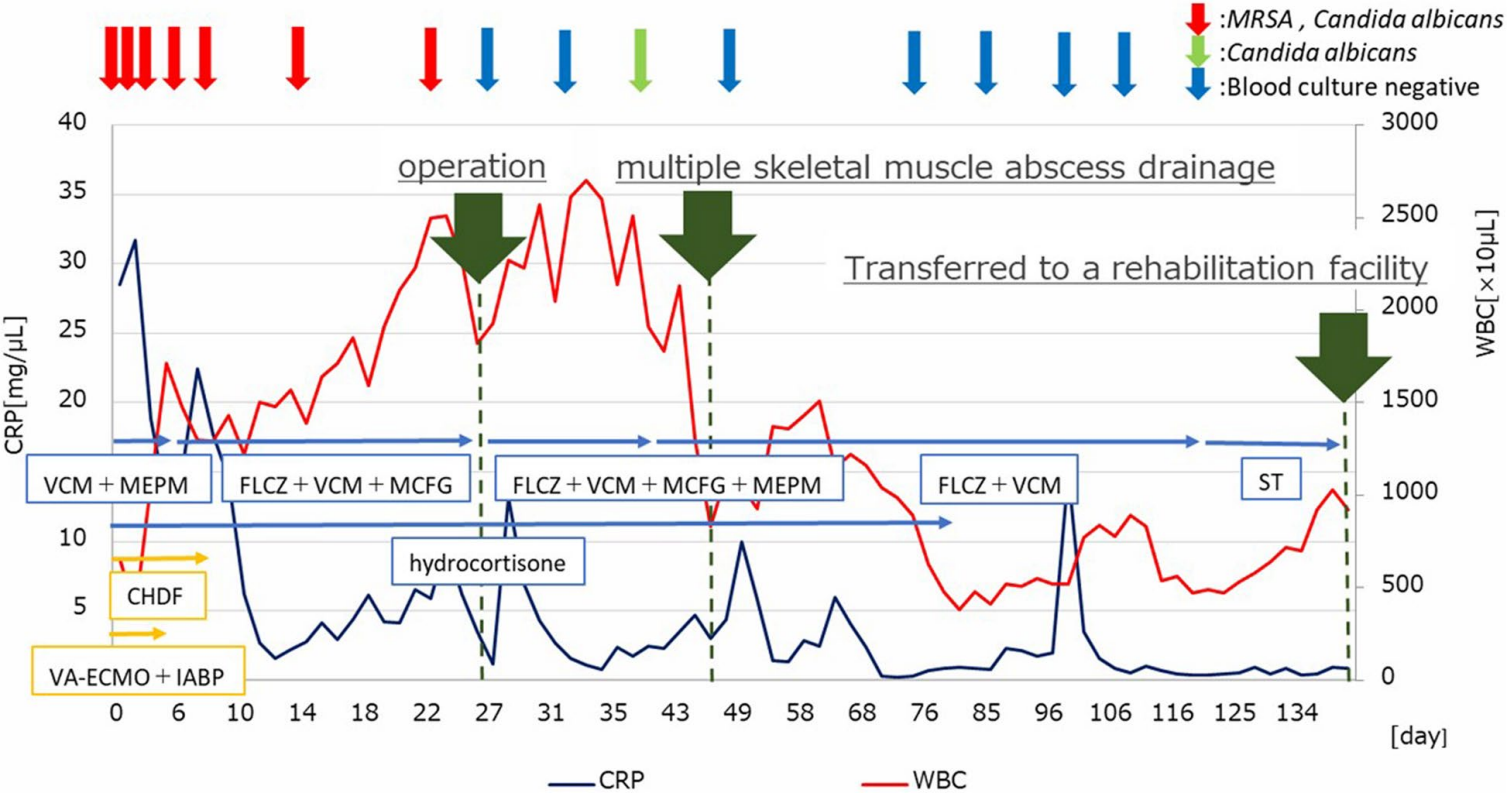
Time course of multiple treatments, weight blood cell counts, serum C-reactive protein level, and the results of blood culture after admission. CHDF, continuous, hemodiafiltration; CRP, C-reactive protein; FLCZ, fluconazole; IABP, intra-arterial balloon pumping; MCFG, micafungin; MEPM, meropenem; MRSA, methicillin-resistant *Staphylococcus aureus*; ST, trimethoprim-sulfamethoxazole; WBC, white blood cell; VA-ECMO, venoarterial extracorporeal membrane oxygenation; VCM, vancomycin

She was diagnosed with infective endocarditis complicated with systemic multiple abscesses due to CA-MRSA and *C. albicans*. However, because of brain hemorrhage and low platelet count, primary surgical treatment was considered intolerable. Therefore, medical antibacterial treatment with fluconazole (FLCZ), meropenem (MEPM), and vancomycin (VCM) was introduced, and heart failure treatment with mechanical circulatory support and renal replacement therapy was continued (Fig. 3). A steroid therapy was also introduced for the destructive thyroiditis. Hemodynamics were stabilized, and ECMO and IABP were weaned off on day 5 of admission. Renal function also normalized, and the renal replacement therapy could also be discontinued. The platelet counts gradually increased and reached 10.0 × 10^4^/µL, and surgical treatments were conducted on day 26 of admission after regression of cerebral hemorrhage was confirmed by brain CT scan (Fig. 2B).

**Fig. 4:**
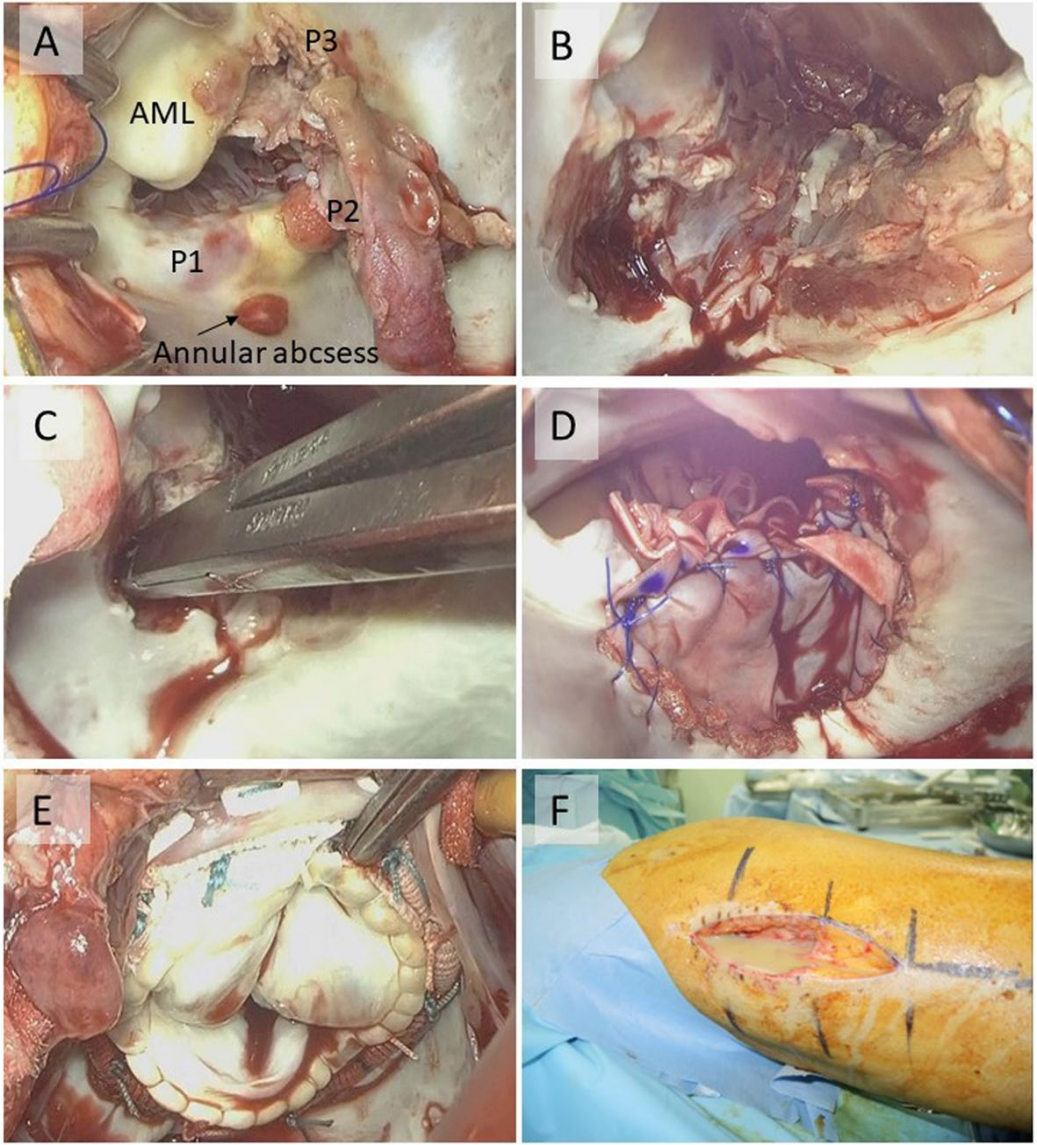
**A** Vegetations were found on all the leaflets of the mitral valve, the annulus of P1 portion, and on subvalvular apparatus. The P2-3 portions of the posterior leaflet were most heavily destructed. AML: anterior mitral leaflet. **B** Both leaflets were resected together with subvalvular apparatus including the bilateral papillary muscles. **C** The P1 annulus was cut open, and the abscess was completely debrided, which resulted in a fistula into which the tip of a forceps advanced for about 1.5 cm. **D** The missing posterior annulus was reconstructed with a bovine pericardial patch. **E** Mitral valve replacement with a biological valve was done. **F** Drainage of recurrent multiple skeletal muscle abscess was performed by orthopedics team 3 weeks after the operation

Prior to cardiac surgery, partial right lung resection was performed under video-assisted thoracic surgery for right lung abscess and secondary pneumothorax by thoracic surgeons. Then, the patient was placed on supine position, and the surgery for infective endocarditis was done through median sternotomy. Opening the pericardium, dense adhesion was encountered. After establishing cardiopulmonary bypass, aorta was cross-clamped, and the right atrium was opened. A large vegetation was attached to the edges of anterior and posterior leaflets of the tricuspid valve, and it continued down to the chordae and the papillary muscle. The vegetation was completely resected together with the edges of leaflets, chordae, and the tip of the papillary muscle. The right heart cavity was rinsed with saline. Then, the mitral valve was approached via a transseptal superior approach. Although the main vegetation was located on P2–3 portion of the posterior leaflet, vegetations were also found on P1 and A2 portion of the mitral valve leaflet, chordae tendinea, and on both papillary muscles (Fig. 4A). Furthermore, annular abscess was observed in the annulus of P1 portion (Fig. 4A). Both leaflets were resected together with subvalvular apparatus including the bilateral papillary muscles (Fig. 4B). The P1 annulus was cut open, and the abscess was completely debrided, which resulted in a fistula at the P1 annulus into which the tip of a forceps advanced for about 1.5 cm (Fig. 4C). After the left heart cavity was meticulously rinsed with saline, the missing posterior annulus was reconstructed with a bovine pericardial patch (Fig. 4D), and mitral valve replacement with a biological valve was done (Fig. 4E). The tricuspid valve was repaired with 2 artificial chordae on anterior and posterior leaflets and ring annuloplasty. Cardiopulmonary bypass could be weaned off without mechanical support, and intraoperative transesophageal echocardiography revealed no mitral regurgitation and mild tricuspid regurgitation. Subsequent to heart surgery, debridement was performed for abscesses in the left and right thighs and right shoulder joints by orthopedics team.

After the operation, the blood culture became negative for the first time during the course and kept being negative except for one occasion when it grew *C. albicans* 2 weeks after the operation (Fig. 3). The postoperative antibacterial treatment consisted of 4 agents with FLCZ, MEPM, micafungin, and VCM, and it was decreased to 2 agents (FLCZ and VCM) 2 weeks after the operation. Despite the negative blood culture, the white blood cell counts remained high, and C-reactive protein remained positive in the blood test (Fig. 3), and contrast-enhanced CT scan revealed recurrence of multiple skeletal muscle abscess. Therefore, the abscess drainage was performed by orthopedics team again 3 weeks after the operation (Fig. 4F). The inflammatory signs in the blood test subsided thereafter, and repeated CT scan revealed no recurrence of the abscess. Postoperative transthoracic echocardiography showed a normal left ventricular contractility with left ventricular ejection fraction of 63%, no vegetation, no paravalvular leakage of the mitral valve, and mild tricuspid regurgitation. Finally, the antibiotics were changed to oral trimethoprim-sulfamethoxazole combination, and the patient was discharged to a rehabilitation facility on 114 days. Brain CT scan before discharge revealed no recurrence of cerebral hemorrhage (Fig. 2C). Genomic analysis of MRSA isolated from blood revealed Staphylococcal Cassette Chromosome mec (SCCmec) IVc containing the *mecA* gene responsible for methicillin resistance and also the virulence gene encoding PVL.

## Discussion and conclusions

We presented a case of infective endocarditis caused by CA-MRSA and *C. albicans* complicated with systemic multiple abscess and disseminated intravascular coagulation. Genomic analysis of the MRSA strain revealed SCCmec type IVc and PVL gene. Although the patient was transferred to our hospital in critically ill condition with cardiogenic shock and uncontrollable infection, we decided not to perform the radical surgery emergently. We delayed the operation for the next reasons: (1) the patient presented with cerebral hemorrhage and full heparinization for cardiopulmonary bypass might cause further bleeding, (2) the patient also presented with disseminated intravascular coagulopathy with low platelet count (2.0 × 10^4^/µL), and intraoperative bleeding could be uncontrollable, and (3) the patient was in cardiogenic shock status with end-organ dysfunction, and it is reported that delayed surgery after stabilization of hemodynamics and improvement in end-organ function yield better outcomes than emergency surgery [3]. Hence, multidisciplinary treatment with antibacterial therapy, steroid therapy, renal replacement therapy, and mechanical circulatory support was required for rescue. The patient was finally successfully treated with multiple surgery including multiple systemic abscess drainage, pneumonectomy, and heart surgery.

Over the past 30 years, MRSA strains have emerged as serious pathogens in the nosocomial and community setting [4]. In Japan, MRSA isolates have been prevailing in academic hospitals since the late 1980s. Their spread in community hospitals was witnessed in the 1990s [5]. This increase in the incidence of MRSA infection has been associated with the recognition of new MRSA clones known as CA-MRSA. This organism is distinct from HA-MRSA in terms of epidemiology, microbiology, and clinical manifestation. CA-MRSA clones involve previously healthy individuals without either direct or indirect association with healthcare facilities and have emerged as a new and important public health problem [6]. It remains unclear as to whether CA-MRSA evolved historically from the acquisition of SCCmec elements conferring methicillin resistance, through an altered penicillin-binding protein (PBP-2′) in the bacterial cell wall, within methicillin-susceptible *S. aureus* in the community or if CA-MRSA was originally derived from HA-MRSA. CA-MRSA has traditionally been distinguished from HA-MRSA by the presence of SCCmec types IV or V (HA-MRSA normally contains SCCmec types I, II, or III) [6]. CA-MRSA strains have higher pathogenicity and are more destructive to tissue than HA-MRSA strains because of the toxins they produce, including PVL [1, 2, 5]. CA-MRSA is primarily associated with skin and soft tissue infections (abscesses, cellulitis, and furunculosis); however, there have been severe cases of PVL producing CA-MRSA infection associated with septic shock, bacteremia, necrotizing pneumonia, and endocarditis [7, 8].

Over the last decade, *S. aureus* has been the leading cause of left-sided infective endocarditis. Now, reports of infective endocarditis attributed to CA-MRSA are emerging in the literature. Most reported cases of infective endocarditis caused by CA-MRSA occurred in adults without valvular anomaly and without any known predisposing factors for infective endocarditis [9, 10]. Preexisting skin lesions and intravenous drug abuse were the major risk factors [7, 8, 10]. In the present report, the patient had no obvious risk factors for infective endocarditis. Although she had small tattoos on her left wrist and on her lower back, there was no skin abnormality. She denied intravenous drug abuse. CA-MRSA may cause native valve endocarditis among non-intravenous drug users and with no preceding infections [10], and this is one of distinct characteristics of CA-MRSA compared to HA-MRSA. Most reported CA-MRSA infective endocarditis cases were, as in our case, PVL positive [7, 10]. PVL is a neutrophilolytic toxin that forms pore-forming toxins that target polynuclear leukocytes and macrophages, thereby weakening the immune system [8, 11]. We considered this explains the extremely invasive feature of the CA-MRSA infection in the present case. It is reported that PVL-positive rate is on the rise in Japan [12].

Another distinctive feature of the present case was the coinfection of *C. albicans* with CA-MRSA. Fungal endocarditis itself is also a rare and fatal condition. It accounts for 1–6% of the total endocarditis spectrum and remains the most serious form of infective endocarditis, with a high mortality rate of about 50% [13]. It also increases the potential risk of congestive heart failure and many other maladies [14]. In particular, the *Candida* species, the most common etiologic fungi found responsible for fungal endocarditis, have been associated with a substantial increase in *in-hospital* morbidity and mortality [13, 14]. Risk factors include prosthetic heart valves, injection drug use, parenteral nutrition, immunosuppression, and broad-spectrum antibiotic regimens [15, 16]. Although none of these risk factors was found in our case, the patient developed *C. albicans* infection. We considered PVL producing CA-MRSA infection was the important factor for developing the *C. albicans* infection in this patient. Coinfections of fungi and bacterium could result in a refractory condition [17], and this was obviously one of the factors that caused the treatment course so complicated in the present case.

In conclusion, we presented a case of infective endocarditis caused by the coinfection of PVL producing CA-MRSA and *C. albicans*. The patient was successfully treated with multidisciplinary treatment including aggressive surgery.

## Data Availability

All data generated or analyzed during this study are included in this published article.

## Abbreviations

*CA-MRSA*: Community-acquired *methicillin-resistant Staphylococcus aureus*
CT: Computed tomography
ECMO: Extracorporeal membrane oxygenation
FLCZ: Fluconazole
*HA-MRSA*: Healthcare-associated *methicillin-resistant Staphylococcus aureus*
IABP: Intra-aortic balloon pumping
MEPM: Meropenem
PVL: Panton-Valentine leukocidin
SCCmec: Staphylococcal Cassette Chromosome mec
VCM: Vancomycin
